# Celebrating the 50^th^ Issue of Qatar Medical Journal: Editorial Letter

**DOI:** 10.5339/qmj.2021.43

**Published:** 2021-12-13

**Authors:** Mohamad Alkadi

**Affiliations:** ^1^Division of Nephrology, Department of Medicine, Hamad General Hospital, Doha, Qatar E-mail: malkadi@hamad.qa; ^2^Weill Cornell Medicine, Doha, Qatar

## Abstract

It is with great pride that we celebrate the 50^th^ issue of Qatar Medical Journal (QMJ) that has achieved significant growth recently. Our mission is to encourage authors to submit high-quality and innovative research promoting medical advancements. In the past two years, manuscripts submissions have tripled in number and were enriched by a more diverse pool of authors with global representation, resulting in an increase in the number of published issues moving from being a biannual to a triannual journal. Additionally, the number of articles published in an issue has doubled. QMJ continues to be an open-access peer-reviewed journal, publishing original research work, reviews, editorials, and case reports that are particularly relevant to medicine and free of charge to authors. It is indexed in several renowned and highly ranked platforms such as PubMed Central, Scopus, Scimago, Google Scholar, and the Directory of Open Access Journals. It was also recently indexed in the World Health Organization's Index Medicus for the Eastern Mediterranean Region (IMEMR). We look forwards to becoming the highest-rated medical journal, in terms of impact factor, regionally.

